# Giant viable hydatid cyst of the lung revealed by hiccups

**Published:** 2012-11-13

**Authors:** Bouchentouf Rachid, Benjelloun Amine, Chafik Aziz, Aitbenasser Moulay Ali

**Affiliations:** 1 Department of pulmonology, Avicenna Military Hospital, Marrakech, Morocco; 2 FMPM, Cadi Ayyad University, Marrakech, Morocco; 3 Department of Thoracic Surgery, Avicenna Military Hospital, Marrakech, Morocco

**Keywords:** Hiccups, giant hydatid cyst, lung, surgery

## Abstract

Hydatid cystic disease is still a significant clinical problem in endemic countries. Hydatid cysts are usually located in the liver and lung. The resulting large cysts in the lung are a special clinical entity called giant hydatid cysts. We present a case of this rare entity: An 18-year-old woman presented with three month history of hiccups and progressive dyspnea. Chest X-ray revealed a very large homogenous opacity of the left lung. A diagnosis of giant hydatid cyst was made intra operatively; the patient was treated surgically using cystotomy and capitonnage without post-operative complications. This report illustrates that the hydatid cyst of the lung may occasionally present with signs of mediastinal compression.

## Introduction

A hydatid cyst is a parasitic infection caused by Echinococcus Granulosus. The liver is the most commonly affected organ (> 65%) followed by the lung (>25%) [1], which is the predominant site of cyst formation in children. We report the case of pulmonary giant hydatid cyst revealed by hiccups and for which we have not found any similar case in the literature.

## Patient and observation

**Figure 1:**
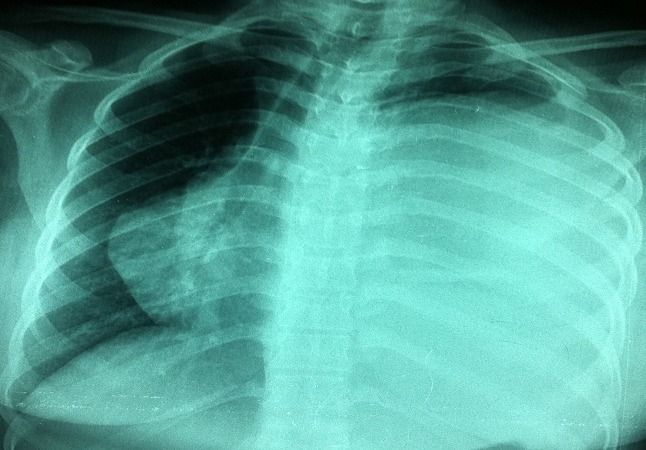
Posteroanterior radiograph of the giant pulmonary hydatid Cyst which pushed the heart to the right side

**Figure 2:**
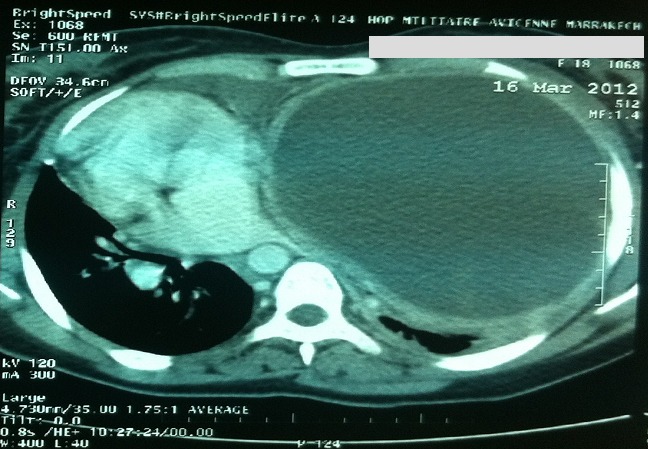
The computed tomography scan demonstrating the cyst completely involved the left Thorax

An 18-year-old woman presented with three month history of hiccups and progressive dyspnea. The patient was otherwise healthy, but there was a history of exposure to domestic dogs and sheep. Pulmonary examination revealed an abolition of breath sounds at auscultation of the left hemi thorax which was dull on percussion. Abdominal examination was normal. Chest radiograph postero-anterior view showed a very large dense homogenous opacity occupying nearly the entire left lung (Figure 1). Sonographic evaluation of the chest revealed a voluminous fluid collection occupying nearly the entire left hemithorax. The greatest collection contains daughter cysts. The computed tomography of thorax revealed a giant cystic lesion (29 cm x 17cm) occupying a large proportion of the left lung and pushing the heart to the right side (Figure 2). Biological investigations revealed the following: haemoglobin: 10 g/dl, white cells: 5700, C Reactive protein: 62 mg/l. Serum immunoglobulin titers were negative for *Echinococcus granulosus*. Abdominal ultrasonography did not revealed cysts. The diagnosis of giant hydatid cyst was made intraoperatively and cyst was removed conservatively. The postoperative was satisfactory and hiccups disappeared completely. Written informed consent was obtained for publication of this case report and accompanying images.

## Discussion

Hiccups are a sudden, irregular and spasmodic contraction of the diaphragm followed by an abrupt closure of the glotti. The cause of hiccups was due to compression of the phrenic nerve in the mediastinum. Echinococcosis is a parasitic disease frequently seen in sheep and cattle raising countries with poor sanitary conditions. Hydatid disease is endemic in Morocco; the annual incidence of chirurgical hydatid disease is 6.4/100000 in the Moroccan population [2]. Hepatic infestation is most commonly seen followed by lung involvement that has been seen in 25% of the cases, in some series [1]. Giant hydatid cysts of the lung are defined as cysts measuring 10 cm or more [3]. This entity is most frequently encountered in adolescents and in children older than ten year [3, 4]. This predominance is explained by the fact the immune system and the relatively higher elasticity of the lung tissue in children and adolescents allows the rapid growth of cysts. The lung hydatid disease is frequently asymptomatic in children [3]. Nevertheless, giant lung hydatid cysts were constantly symptomatic [3–5]. Common presenting symptoms of giant pulmonary hydatid cyst are compression symptoms such as dry cough, while ruptured cyst can cause productive cough, chest pain, dyspnea, and very rarely anaphylactic shock. In our case, symptoms were chest pain and dyspnea, and hiccups. The diagnosis is easy in endemic areas. The patient is usually in good general health in cases of non-complicated cysts and chest X-ray will show a well-circumscribed dense homogenous opacity [6]. Giant pulmonary hydatid cysts can be located elsewhere in the lung but in literature they were located most commonly in the right lower lobe. In our case hydatid cyst was located in the left lower lobe. Computed tomography (CT) scan is not mandatory; however, it displays better information about the size of the hydatid cyst, and its relation to the lung parenchyma [7]. Serological tests have limited diagnostic value, and the inclusion of abdominal ultrasonography in the protocol of investigation is necessary because of the coexistence of hepatic cysts [8].

The chest X-ray and computerized thoracic tomography are generally sufficient for diagnosis. However a definite diagnosis was based on pathological confirmation. Surgery remains the treatment of choice for hydatid disease; the current treatment is complete excision of disease process with maximum preservation of the lung tissue. Medical treatment can be used during the postoperative period, in order to prevent the recurrence and occurrence of a secondary cyst.

## Conclusion

Pulmonary giant hydatid cyst is a rare and a special clinical entity. The diagnosis must be evoked in front of opacity of the hemi thorax especially in a country of endemic disease.
